# From clinical appearance to accurate management in acute ischemic stroke patients: With the guidance of innovative traditional Chinese medicine diagnosis

**DOI:** 10.1002/brb3.1411

**Published:** 2019-09-30

**Authors:** Juexian Song, Cuiyu Xu, Jing Zhang, Li Gao

**Affiliations:** ^1^ Department of Neurology Xuanwu Hospital Capital Medical University Beijing China; ^2^ Department of Emergency Beijing Hospital of Traditional Chinese Medicine Capital Medical University Beijing China

**Keywords:** acute ischemic stroke, guidance, integrated Chinese and Western medicine, laboratory indicators, simplified classification

## Abstract

**Objective:**

To investigate the correlation between simplified classification and laboratory indicators in patients with acute ischemic stroke, also provide accurate evidences for simplified classification and guide clinical interventions and treatment.

**Methods:**

Two hundred patients with acute ischemic stroke were classified into four types according to the characteristics of Traditional Chinese Medicine syndrome: phlegm‐heat syndrome, phlegm‐dampness syndrome, qi deficiency syndrome, and yin deficiency syndrome. The differences between the types of syndromes and the correlation between laboratory indicators and syndromes were analyzed.

**Results:**

Among the 200 patients with acute ischemic stroke, there were significant differences in the level of low‐density lipoprotein (LDL‐C) (*p* < .05) between patients with phlegm‐heat syndrome and other three types. There were significant differences in the levels of homocysteine (HCY) and fibrinogen (Fib) between patients with yin deficiency syndrome and other three types (*p* < .05). In addition, there were statistically significant differences in blood glucose (Glu), glycosylated hemoglobin (HBA1c), and total cholesterol (CHO) between phlegm‐heat syndrome and qi deficiency syndrome (*p* < .05). There were significant differences in the levels of Glu, HBA1c, D‐2 polymer (D‐D), and C‐reactive protein (CRP)s between patients with phlegm‐heat syndrome and phlegm‐dampness syndrome (*p* < .05). There were statistically significant differences in the levels of CRP and urea nitrogen between patients with yin deficiency syndrome and phlegm‐dampness syndrome and qi deficiency syndrome (*p* < .05).

**Conclusions:**

The four‐type simplified classification of Integrated TCM and Western medicine in acute ischemic stroke has specific laboratory data to support. Simplified classification with TCM treatment and intervention of different patients improves the survival and treatment, which is an innovative, easy‐to‐master clinical diagnosis and treatment model.

## Graphical abstract

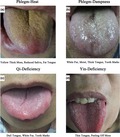

## 1 INTRODUCTION

Acute ischemic stroke is one of the major disabling and lethal diseases in the world that has become the first cause of death in China with its increasing incidence and younger trend (Chen, 2008). At present, Western medicine treatment is mainly based on basis such as thrombolysis, anti‐platelet aggregation, lipid‐lowering, spots stabilization, blood pressure control, and blood glucose control, which lacks individualized treatment plans and new treatment ideas. The disease belongs to the category of “stroke disease” in traditional Chinese medicine, with standardization of syndromes and clinical application are attracting more and more attention (Zhao, Zhang, & Li, 2018). Therefore, simplifying syndrome differentiation is especially important for clinical diagnosis and treatment. An in‐depth study of TCM syndromes and integrated into Western diagnostics has been proposed, leading to a new medical model diagnosis and treatment program (Jiang et al., 2012).

At present, the most widely used stroke classification is Trial of Org 10172 in Acute Stroke Treatment (TOAST) with a total of five subtypes: (a) large‐artery atherosclerosis, (b) cardioembolism, (c) small‐vessel occlusion, (d) stroke of other determined etiology, and (e) stroke of undetermined etiology. There are also researches on TCM syndrome types. Based on years of clinical practice, the team of integrated Chinese and Western medicine in the Department of Neurology of Xuanwu Hospital has simplified the classification of acute ischemic stroke into four types: phlegm‐heat syndrome, phlegm‐dampness syndrome, qi deficiency syndrome, and yin deficiency syndrome, on which condensed the prescriptions of the Chinese medicine agreement corresponding to the syndrome type: Tanhuofang, Tanshifang, Qixufang, and Yinxufang, which have achieved good clinical results. The correlation between the simplified classification and laboratory indicators will provide objective evidence for the classification and provide new ideas for the management of patients with acute ischemic stroke and also promote the new diagnosis and treatment models. Simplified classification and laboratory indicators analyzing of 200 patients with acute ischemic stroke admitted from February 2017 to December 2017 in Department of Neurology, Xuanwu Hospital of Capital Medical University are reported as follows.

## 2 MATERIALS AND METHODS

### 2.1 Patients

After permission of Ethics Committee of Xuanwu Hospital, Capital Medical University, 200 patients with acute ischemic stroke were analyzed and simplify classified according to the TCM characteristics of syndromes. The TOAST classification of 200 patients belonged to the large‐artery atherosclerosis type. All patients signed informed consent before enrollment.

### 2.2 Traditional Chinese medicine acute ischemic stroke syndrome (Xuanwu classification)

The classification of phlegm‐heat syndrome, phlegm‐dampness syndrome, qi deficiency syndrome, and yin deficiency syndrome was depending on the TCM characteristics of syndromes (Table 1).

**Table 1:** Traditional Chinese medicine acute ischemic stroke syndrome

Syndrome	Characters
Phlegm‐heat syndrome	Yellow thick moss, reduced saliva, dry mouth, bitter mouth, sticky mouth, thirsty for cold drinks, have bad breath, fear of heat, dry stool, big smell
Phlegm‐dampness syndrome	White fur, moist, thick tongue, tooth marks, pale mouth, thirsty without the want to drink, hands and feet not warm, stool sticky
Qi deficiency syndrome	Dull tongue, white fur, can have tooth marks, sweating more, afraid of cold, dry and light mouth, four ends not warm, loose stools or poor bowel movements
Yin deficiency syndrome	Thin tongue, peeling off moss, dark red tongue, hot flashes, night sweats, thirst for cold drink

### 2.3 Inclusion criteria

(a) onset conforms to the diagnostic criteria established by the Fourth National Conference on Cerebrovascular Diseases; (b) Aged between 35 and 75 years old; (c) 1 point ≤ National Institute of Health stroke scale(NIHSS) score ≤ 20 points; (d) Head CT or MRI was performed within 72 hr after admission with a localized high signal in the T2‐weighted image and a high signal in the DWI.

### 2.4 Exclusion criteria

(a) Suffering from serious systemic diseases that may affect the outcome, such as liver and kidney disease, blood system diseases, autoimmune diseases, malignant tumors, chronic serious infections, mental illness, drug abuse, alcoholics, and so on. (b) Poor fit and poor compliance.

### 2.5 Research methods

In all cases, blood and urine samples were collected with an empty stomach condition on the next morning of the hospitalization, and laboratory indicators were collected. Biochemical series: blood glucose (Glu), high‐density lipoprotein (HDL‐C), low‐density lipoprotein (LDL‐C), total cholesterol (CHO), triglyceride (TG), apolipoprotein A (Apo‐A), apolipoprotein B (Apo‐B), creatinine (Cr), uric acid (UA), urea nitrogen (UREA). Coagulation series: D2 polymer (D‐D), fibrinogen (Fib), activated partial thrombin time (APTT), international normalized ratio (INR), clotting time % (PT%). Thyroid function: thyrotropin (TSH), triiodothyronine (T3), thyroxine (T4), free triiodothyronine (FT3), free thyroxine (FT4). Others: glycated hemoglobin (HbA1c), C‐reactive protein (CRP), homocysteine (HCY), folic acid (FOL), vitamin B12 (VB12), urine PH (Urine PH).

According to the characteristics of syndromes, the symptoms of each patient were objectively recorded and simplified. The specific descriptions included facial color, tongue image, onychomycosis, high and low sound, bad breath, defecation, sweating, the preference of drinking and diet, and the temperature of hands and feet. The significant differences between the various laboratory indicators in the four types of syndromes were analyzed.

### 2.6 Statistical methods

The data were sorted by Microsoft Excel 2010 and processed and analyzed by SPSS 17.0. The continuous variables were expressed by $\bar{\chi }\pm s$, and the *t* test was used for comparison. The classified variables were expressed as percentage, and the chi‐square test was used for comparison. *p* < .05 was considered statistically significant.

## 3 RESULTS

### 3.1 General information

**Figure 1:**
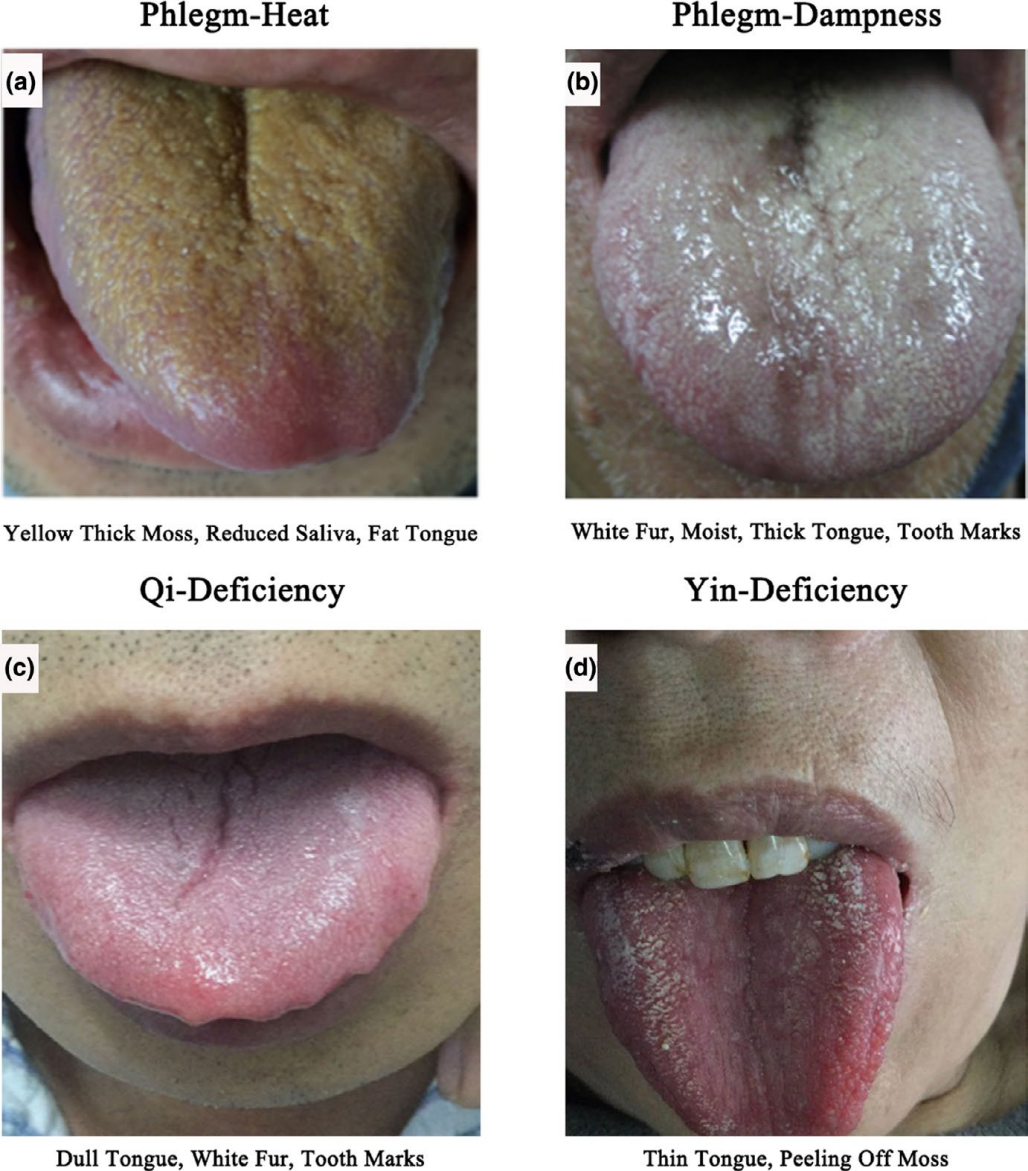
The tongue appearance of four types

There were 138 male patients and 62 female patients. The ratio of male to female was 2.2:1, with an average age of 57 ± 10.1 years, including 130 cases with phlegm‐heat syndrome (65%), 35 cases with phlegm‐dampness syndrome (17.5%), and 30 cases with qi deficiency syndrome (15%), 5 cases of yin deficiency syndrome (2.5%). There were 113 patients with hypertension, including 84 patients with phlegm‐heat syndrome (64.6%), 17 patients with phlegm‐dampness syndrome (48.6%), 8 patients with qi deficiency syndrome (26.7%), and 4 patients with yin deficiency syndrome (80%). The main tongue appearance showed in Figure 1. There were 65 patients with type 2 diabetes, including 49 patients with heat syndrome (37.7%), 11 patients with phlegm syndrome (31.4%), 4 patients with qi deficiency syndrome (13.3%), and 1 patient with yin deficiency syndrome (20%). There were 75 patients with hyperlipidemia, including 52 patients with phlegm‐heat syndrome (40%), 15 patients with phlegm‐dampness syndrome (42.9%), 6 patients with qi deficiency syndrome (20%), and 2 patients with yin deficiency syndrome (40%). There were 15 patients with coronary heart disease, including 10 patients with phlegm‐heat syndrome (7.7%), 1 patient with phlegm‐dampness syndrome (2.9%), 4 patients with qi deficiency syndrome (13.3%), and no patient with yin deficiency syndrome (0%). There were 33 patients with old ischemic stroke, including 24 patients with phlegm‐heat syndrome (18.5% of patients with phlegm‐heat syndrome), 2 patients with phlegm‐dampness syndrome (5.7%), 6 patients with qi deficiency syndrome (20%), and 1 patient with yin deficiency syndrome (20%; Table 2).

**Table 2:** Basic information of all patients

	Phlegm‐heat	Phlegm‐Dampness	Qi deficiency	Yin deficiency	*p* value
Cases	130	35	30	5	
Portion (%)	65	17.5	15	2.5	<.001
Gender					
M	88	20	21	3	.104
F	42	15	9	2	
Average age	58.08 ± 10.22	54.93 ± 9.55	54.92 ± 7.94	63.2 ± 11.8	.083
Hypertension	84	17	8	4	.031
Diabetes	49	11	4	1	.067
Hyperlipidemia	52	15	6	2	.109
Coronary heart disease	10	1	4	0	.023
Old cerebral infarction	24	2	6	1	.027
NIHSS	5.88 ± 3.16	3 ± 3	2.85 ± 3.66	5.2 ± 3.92	.036
mRS	1.56 ± 1.01	1.91 ± 1.08	1.69 ± 1.26	2.2 ± 1.47	.385

### 3.2 Laboratory indicators

Tables 3, 4, 5, 6 show the average value of the laboratory parameters (including biochemical series, coagulation series, thyroid function series, and other indicators) of 200 patients in four syndrome types. The normal levels of all parameters are as follows: Glu: 3.89–6.1 mmol/L; HbA1c: 4%–6%; TCHO:3.5–6.1 mmol/L; TG: 0.22–1.21 mmol/L; HDL‐C: 0.91–2.19 mmol/L; LDL‐C: <3.12 mmol/L; Apo‐A: 1–2 g/L; Apo‐B: 0–1 g/L; CR: male: 44–133 μmmol/L; female: 70–106 μmmol/L; UREA: 2.0–7.1 mmol/L; UA: male:150–416 μmol/L, female: 89–357 μmol/L；D‐D: 0.2 μg/ml; FIB: 2–4 g/L; APTT: 23–38 s; INR: 0.8–1.5; PT%: 70–130; TSH: 2–10 mU/L; T3: 1.34 ~ 2.73 nmol/L; T4 :78.4 ~ 157.4 nmol/L; FT3: 2.0–6.6 pmol/L; FT4: 0.8–2.3 ng/dl; FOL: ≥6.8 nmol/L; VB12: 630–5300 pg/ml; HCY: 5–15 μmol/L; CRP: 0–10 mg/L; Urine PH:5–8.5. In patients with phlegm‐heat syndrome, Glu, HbA1c, and D‐D were above the normal level, and HDL‐C was lower than the normal level. In patients with phlegm‐dampness syndrome, the mean HDL‐C was below the normal range, and the remaining laboratory indicators were within the normal range. In patients with qi deficiency syndrome, the levels of HDL‐C and LDL‐C were lower than the normal level. In patients with yin deficiency syndrome, Glu, HbA1c, D‐D, FIB, HCY, and CRP were higher than normal values, and TCHO, HDL‐C, and LDL‐C were lower than the normal reference value range. See the following table for details. The *p* value of the laboratory indicators of 200 patients tested between different syndrome types.

**Table 3:** The comparison of biochemistry indicators ($\bar{\chi }\pm s$)

biochemistry series	Phlegm‐heat	Phlegm‐Dampness	Qi deficiency	Yin deficiency
Glu (mmol/L)	7.17* ± 3.04	5.77 ± 1.63	5.68 ± 1.51	6.79* ± 1.82
HbA1c (%)	6.99* ± 1.90	5.99 ± 0.85	5.88 ± 0.86	6.04* ± 0.91
TCHO (mmol/L)	4.17 ± 1.02	3.82 ± 1.19	3.58 ± 1.02	3.13* ± 0.45
TG (mmol/L)	1.70 ± 0.90	1.70 ± 1.24	1.47 ± 0.57	1.48 ± 0.43
HDL‐C (mmol/L)	1.03* ± 0.26	0.98* ± 0.24	1.06* ± 0.27	0.85* ± 0.11
LDL‐C (mmol/L)	2.70 ± 0.87	2.27 ± 1.01	2.06* ± 0.78	1.81* ± 0.44
Apo‐A (g/L)	1.17 ± 0.22	1.10 ± 0.19	1.10 ± 0.21	0.96 ± 0.14
Apo‐B (g/L)	0.84 ± 0.24	0.73 ± 0.28	0.68 ± 0.22	0.65 ± 0.10
CR (μmol/L)	68.82 ± 25.25	74.15 ± 19.25	71.41 ± 21.37	67.4 ± 17.56
UREA (mmol/L）	4.99 ± 1.74	4.47 ± 1.09	4.23 ± 1.51	6.19 ± 2.02
UA (μmol/L)	301.61 ± 92.01	315.52 ± 80.35	302.48 ± 80.04	284.2 ± 116.25

* *p*: .05

**Table 4:** The comparison of coagulation indicators ($\bar{\chi }\pm s$)

Coagulation	Phlegm‐heat	Phlegm‐Dampness	Qi deficiency	Yin deficiency
D‐D (μg/ml)	0.86* ± 1.38	0.42 ± 0.48	0.40 ± 0.28	0.81* ± 0.67
FIB (g/L)	3.32 ± 0.91	3.25 ± 0.74	3.22 ± 0.80	4.70* ± 0.56
APTT (s)	36.85 ± 3.98	36.61 ± 3.34	37.18 ± 4.49	34.16 ± 2.83
INR (INR)	1.03 ± 0.12	1.01 ± 0.08	1.03 ± 0.05	1.03 ± 0.06
PT% (%)	97.55 ± 14.96	99.23 ± 14.19	96.33 ± 8.37	96.2 ± 9.58

* *p*: .05

**Table 5:** The comparison of thyroid function indicators ($\bar{\chi }\pm s$)

Thyroid function	Phlegm‐heat	Phlegm‐Dampness	Qi deficiency	Yin deficiency
TSH (mU/L)	2.09 ± 1.92	2.34 ± 1.67	2.35 ± 1.41	2.64 ± 2.70
T3 (nmol/L)	0.99 ± 0.19	1.03 ± 0.14	1.09 ± 0.15	0.90 ± 0.17
T4 (nmol/L)	8.41 ± 1.80	8.67 ± 1.88	8.54 ± 2.71	8.7 ± 1.50
FT3 (pmol/L)	2.80 ± 0.38	3.02 ± 0.40	3.10 ± 1.70	2.56 ± 0.20
FT4 (ng/dL)	1.23 ± 0.19	1.29 ± 0.32	1.23 ± 0.42	1.28 ± 0.19

**Table 6:** The comparison of other indicators ($\bar{\chi }\pm s$)

Other indicators	Phlegm‐heat	Phlegm‐Dampness	Qi deficiency	Yin deficiency
FOL (ng/ml)	8.01 ± 4.40	7.13 ± 4.35	6.89 ± 2.86	8.37 ± 5.65
VB12 (pg/ml)	319.12 ± 250.80	340.28 ± 180.41	393.45 ± 194.09	280.8 ± 89.02
HCY (μmol/L)	14.42 ± 6.81	15.84 ± 6.92	14.33 ± 7.23	25.35* ± 18.76
CRP (mg/L)	5.05 ± 5.86	1.65 ± 1.37	2.12 ± 2.55	10.03* ± 5.34
Urine PH	6.13 ± 0.61	6.14 ± 0.57	5.98 ± 0.52	6.88 ± 0.22

* *p*: .05

Table 7 shows the comparison of the laboratory index between the four syndrome types. There were statistically significant differences in the content of LDL‐C between the patients with phlegm‐heat syndrome and other type 3 (*p* < .05), in the content of HCY and Fib between the patients with yin deficiency syndrome and other type 3 (*p* < .05), in Glu, HBA1c and CHO between phlegm‐heat syndrome and qi deficiency syndrome (*p* < .05), in Glu, HBA1c, D‐D, and CRP levels between patients with phlegm‐heat syndrome and phlegm‐dampness syndrome (*p* < .05), and in CRP and BUN between patients with yin deficiency syndrome and phlegm‐dampness syndrome and qi deficiency syndrome (*p* < .05). The specific *p* values are as follows.

**Table 7:** Comparison of the laboratory indicators between the four syndrome types

	PH‐PD	PH‐QD	PH‐YD	PD‐QD	PD‐YD	QD‐YD
HCY	0.355	0.996	0.004*	0.480	0.019*	0.007*
FOL	0.194	0.282	0.869	0.917	0.486	0.529
CRP	0.026*	0.117	0.065	0.829	0.005*	0.012*>
D‐D	0.043*	0.096	0.929	0.955	0.499	0.486
Fib	0.680	0.584	0.001*	0.917	0.001*	0.001*
APTT	0.777	0.700	0.141	0.602	0.209	0.122
INR	0.458	0.870	0.995	0.652	0.751	0.948
PT%	0.581	0.684	0.834	0.456	0.661	0.985
TT	0.108	0.297	0.257	0.659	0.718	0.543
HDL‐L	0.354	0.603	0.122	0.263	0.295	0.095
LDL‐L	0.022*	0.001*	0.025*	0.363	0.269	0.554
CHO	0.108	0.008*	0.030*	0.414	0.181	0.377
TG	0.997	0.238	0.607	0.358	0.629	0.975
APO‐A	0.107	0.089	0.027*	0.928	0.168	0.185
APO‐B	0.041*	0.004*	0.104	0.477	0.527	0.818
Cr	0.296	0.612	0.897	0.676	0.565	0.732
VB12	0.634	0.114	0.677	0.366	0.546	0.259
Urine	0.954	0.255	0.015*	0.346	0.022*	0.006*
PH PT	0.471	0.915	0.931	0.632	0.693	0.899
Glu	0.015*	0.011*	0.757	0.903	0.440	0.402
HBA1c	0.006*	0.004*	0.215	0.812	0.954	0.845
BUN	0.141	0.059*	0.114	0.742	0.035*	0.022*
UA	0.467	0.724	0.286	0.776	0.185	0.250
TSH	0.547	0.540	0.512	0.975	0.729	0.745
T3	0.418	0.081	0.422	0.435	0.268	0.122
T4	0.543	0.759	0.744	0.829	0.971	0.873
FT3	0.155	0.067	0.465	0.700	0.189	0.128
FT4	0.352	0.968	0.695	0.462	0.964	0.702

Abbreviations: PD, Phlegm‐dampness; PH, Phlegm‐heat; QD, Qi deficiency; YD, Yin deficiency.

* *p*: .05

## 4 DISCUSSION

The four‐type simplified classification syndrome characteristics of TCM in acute ischemic stroke were as described above. In the classification. Phlegm‐heat syndrome: yellow thick moss, reduced saliva, dark and fat tongue. Phlegm‐dampness syndrome: white fur, moist, thick tongue, tooth marks. Qi deficiency syndrome: dull tongue, white fur, can have tooth marks. Yin deficiency syndrome: thin tongue, peeling off moss, dark red tongue. Combined with the patient's urine and stools, diet and other conditions, a comprehensive judgment will be made. This four‐type method is  very useful in clinical applications and can effectively help to make comprehensive judgments on patients, which is innovative. In the classification of syndrome type, tongue diagnosis plays an important role (Liu et al., 2014), and the characteristics of tongue images are as follows.

Modern research shows that cerebral arteriosclerosis is the pathological basis of cerebrovascular disease, while lipid metabolism disorder, hypercoagulable state, and vascular inflammatory injury are keys to the occurrence of cerebral arteriosclerosis (Ding, 2010). The above analysis is on the syndrome characteristics of the four types. Exploring the specific laboratory indicators which related  to the four types to provide an objective laboratory basis for the syndrome classification helps to objectively judge the syndrome type and to correlate the pathological basis, enriching the theoretical content of Integrative Chinese and Western Medicine.

In this study, it is found that the proportion of patients with phlegm‐heat syndrome was the highest (65%), whose level of LDL‐L was higher than that of other type 3 with statistically significance (*p* < .05). Ischemic stroke is a complex disease in which large atherosclerotic (LAA) cerebral infarction is considered to be the most relevant stroke subtype of blood lipids. LDL‐L is one of the important factors in the formation of atherosclerosis with most of the guidelines for the prevention of ischemic stroke aimed at lowering LDL‐L levels (Shi, Wang, & Wang, 2013). There are traditional Chinese medicine studies on lipid‐lowering mainly using methods such as Shengqing Jiangzhuo with Chinese herbal medicines such as red yeast, rhubarb, hawthorn and Polygonum multiflorum (Pang, Zhao, & He, 2016). There are statistically significant differences in Glu and HBA1c levels between patients with phlegm‐heat syndrome and phlegm‐dampness syndrome and qi deficiency syndrome, and in D‐D and CRP levels between patients with phlegm‐heat syndrome and phlegm‐dampness syndrome. Some studies have found that patients undergoing whole cerebral angiography (DSA) experience varying degrees of inflammatory response, manifested as elevated levels of CRP, Fib, and so on, with clinical manifestations of phlegm‐heat syndrome. The intervention group was treated with Tanhuo prescription on the basis of conventional treatment in the control group, whose serum CRP and Fib were significantly decreased, while the indexes of the control group were increased, which indicated that the prescription has certain anti‐inflammatory, anti‐platelet, and fibrin‐lowering effects. It has been confirmed by basic researches (Gao, Wang, & Liu, 2008; Liu, Luo, & Gao, 2009; Xu, Gao, & Song, 2010). The main components of the Tanhuo prescription are forsythia, rhubarb, Coptis, and scutellaria. According to the theory of traditional Chinese medicine, it has the effects of clearing away heat and removing phlegm. Professor Xiaolin Tong has suggested that Chinese herbal medicine Coptis has a certain hypoglycemic effect (Gu, Wang, & Pang, 2017), which after the prescription still requires further studies. After clinical syndrome classification, more attention should be paid to the inflammatory index, blood glucose, glycosylated hemoglobin, and low‐density lipoprotein level, and more strict management of its hypoglycemic and lipid‐lowering drugs should be carried out.

According to the traditional Chinese medical concept, the correlation between qi deficiency and phlegm‐dampness is high, and qi deficiency ends up in phlegm‐dampness. Ji Wang found that patients with phlegm‐dampness syndrome have four up‐regulated genes and six down‐regulated genes, who are more susceptible to diseases such as hypertension and diabetes that increase the risk of stroke (Wang et al., 2013). Studies have shown that gastrointestinal dysfunction leads to folic acid and vitamin absorption disorders induced high HCY, which in turn leads to atherosclerotic diseases including cerebrovascular disease. Other studies have shown that folic acid and vitamin B family do not directly improve cognitive impairment in the elderly, but reduce the incidence of cardiovascular and cerebrovascular diseases by reducing HCY levels (Sacco et al., 2006). In this study, there was no correlation between folic acid, vitamin B12 content and HCY content. Although there was no statistically significant difference in folic acid between patients with qi deficiency syndrome and other three types, the average level of folic acid in patients with qi deficiency was significantly lower than that of the other three syndromes, which provides some reference value for the clinic. Modern research believes that oral administration of folic acid and vitamin B12 drugs reduces the level of HCY to reduce the incidence of cerebrovascular disease, which has preventive significance (Liu et al., 2014). A previous study randomly divided 206 patients into observation group and control group with patients in the observation group given folic acid tablets (5 mg qd) orally, whose HCY levels were significantly decreased. After 12 months of follow‐up, the incidence of stroke and myocardial infarction in the control group were 12.62% and 2.91%, respectively, which were significantly higher than 1.94% and 0.97% of the observation group (*p* < .05; Zhao et al., 2018). For patients with qi deficiency syndrome, clinical attention should be paid to their gastrointestinal absorption function and folic acid level. For patients with phlegm‐dampness syndrome, it still requires further research on their correlation indicators.

Among the 200 patients, only five had yin deficiency syndrome, whose proportion (2.5%) was the least. In order to promote the concept and method of simplifying classification, Professor Li Gao has divided the syndrome of “Yin and Yang as the general outline” according to the eight‐character syndrome of Chinese medicine, and combined with years of clinical experience to divide the ischemic stroke into two types: heat syndrome and non‐heat syndrome, and found two major types of patients. There is indeed an essential difference in the expression of microRNAs (miRNAs; Huang & Han, 2013), and syndromes of phlegm‐heat and yin deficiency can be classified as heat syndrome. In the study, the levels of HCY and Fib in patients with yin deficiency syndrome were significantly higher than those in other types, and there were statistical differences in HCY and Fib levels. Patients with yin deficiency syndrome and phlegm‐dampness syndrome and qi deficiency syndrome also had statistically significant differences in CRP and BUN levels, and their CRP and BUN levels were higher than those of other types. Although the number of cases studied was small, the CRP values of the five patients were higher than the normal level, and the D‐D and Fib levels were also higher than the normal level. In comparison, it was found that the phlegm‐heat syndrome and yin deficiency syndrome were higher than the phlegm‐dampness syndrome and qi deficiency syndrome in the CRP and D‐D levels, which coincided with the above conclusions. Although the number of cases is small, clinical attention should be paid to the inflammatory indicators of patients with yin deficiency syndrome. Studies have shown that the use of phlegm‐reducing Chinese medicine improves the clinical symptoms and reduces the degree of neurological damage by reducing CRP levels (Zhang, 2018).

The conclusion of this study is that partial specific laboratory indicators of cerebrovascular disease syndrome can be used as an important reference for simplified classification. Attention should be paid to the inflammatory index, blood sugar, blood lipid level of patients with heat syndrome, to the folic acid level of patients with qi deficiency syndrome, and to the inflammatory index of patients with yin deficiency syndrome. This study also had limitations. As this was a single center, and small scale study, this might cause higher selection biases, and we cannot establish a stable model through performing multivariate logical regression. Therefore, in the future, larger scale further studies are still needed to clarify these problems.

Under the premise of clear diagnosis of acute ischemic stroke in modern medicine, the study draws on the theory of TCM syndrome differentiation and combines the clinical experience accumulated by the Department of neurology, Xuanwu Hospital for many years to classify acute cerebrovascular disease into four types and explore possible laboratory markers. In theory, it will be objective and feasible to guide the individualized diagnosis and treatment of cerebrovascular diseases with the concepts of traditional Chinese medicine and the theory of syndrome differentiation.

## CONFLICT OF INTEREST

None declared.

## Data Availability

The data that support the findings of this study are available on request from the corresponding author. The data are not publicly available due to privacy or ethical restrictions.
